# Unpacking Digital Dashboards’ Influence on Preventive Health Behavior Among Young Adults †

**DOI:** 10.3390/healthcare13111279

**Published:** 2025-05-28

**Authors:** Georgiana Craciun, Aimee A. Kane, Jacqueline C. Pike

**Affiliations:** Palumbo-Donahue School of Business, Duquesne University, Pittsburgh, PA 15282, USA; kanea@duq.edu (A.A.K.); gerberj3@duq.edu (J.C.P.)

**Keywords:** dashboard, visualization, preventive health behavior, psychosocial beliefs, norms, risk, theory of planned behavior, COVID-19, young adults

## Abstract

**Introduction:** The COVID-19 pandemic highlighted the need for digital tools that support public health decision-making and behavior change. Dashboards became a primary method for communicating infectious disease data. However, their influence on preventive health behaviors (PHBs) is not well understood—especially among young adults. This group is less likely to adhere to PHBs, but highly familiar with online tools. **Methods:** Two experimental studies were conducted with young adult participants (200 in Study 1, 228 in Study 2) who viewed the same COVID-19 data in dashboards with or without actionable components. Participants were randomly assigned to different dashboard conditions to measure, on seven-point Likert scales, their PHB intentions and perceptions of behavioral control, attitudes, norms, and risk. The actionable dashboard interventions, designed using the theory of planned behavior, included dynamic behavioral guidance and risk level visualizations. **Results:** Actionable dashboards versus basic dashboards significantly increased PHB intentions (*B* = 0.84, *p* < 0.001, Study 1). Dynamic behavioral guidance was the key dashboard component influencing PHB intentions (*B* = 0.61, *p* = 0.005, Study 2). Parallel mediation analysis testing norms, attitudes, behavioral control, and perceived risk against one another found that only norms explained the link between the dashboard intervention and PHB intentions (B_boot_ = 0.18 and 0.19). **Conclusions:** Findings suggest that actionable dashboards can effectively promote PHB by influencing psychosocial beliefs. These dashboards provide context and guidance, making risky situations more manageable and directing individuals to appropriate preventive actions. Public health professionals should consider incorporating behavioral guidance into community health dashboards to improve their effectiveness.

## 1. Introduction

Infectious disease outbreaks, such as the coronavirus disease, require individuals to significantly change their lifestyles by limiting social interactions and avoiding crowds [1,2,3]. As more people seek health information online [4,5], digital tools play a vital role in conveying needed behavioral changes. During the COVID-19 pandemic, dashboards provided case data to help individuals make informed decisions about their actions. Increasing case numbers in one’s community suggest limiting social interactions, while decreasing cases may indicate it is safe to resume normal social activities. Timely and comprehensive information from dashboards can help the public navigate pandemics [6]. However, interpreting disease outbreak data can be challenging, as individuals often find it difficult to understand the associated risk level and the appropriate preventive health behavior (PHB) response.

Evidence from COVID-19 and other outbreaks shows that young adults are less likely than other age groups to engage in PHBs [7,8]. At the same time, they are digital natives who frequently use online tools like dashboards, especially in university settings [9]. During the COVID-19 pandemic, many students used campus dashboards. These dashboards varied widely: some displayed only case counts, while others also provided risk levels and behavioral recommendations [10]. The “We Rate COVID Dashboards” project highlighted this variation and underscored the need for research on their effectiveness as a health communication tool [11]. Few of the subsequent studies offer theory-based frameworks to explain why certain dashboards are more effective at engaging users and none examine their influence on PHBs [12,13,14].

Behavioral change is a complex psychosocial phenomenon. Decades of research on PHBs, such as healthy eating, reveal that people are more likely to adopt a behavior when they evaluate it positively, believe others are doing it, and think they can do it [15]. These factors are central to several health promotion theories, including the theory of planned behavior (TPB) [16], the health belief model [17], and social cognitive theory [18]. Recent studies have linked TPB psychosocial beliefs with COVID-19 PHBs, such as social distancing [19,20,21]. However, these correlational studies do not address how health communication influences psychosocial beliefs or identify which matter more for PHBs.

The current research examines how dashboard designs influence young adults’ psychosocial beliefs and behavioral intentions. It responds to calls for digital interventions that promote PHBs among young adults [8,10,19]. We begin by developing a conceptual model linking more (vs. less) actionable dashboards to PHBs via psychosocial beliefs. Next, we present an overview of our experimental approach, which utilizes random assignment to help establish causality. Study 1 compares actionable dashboards to basic dashboards. Study 2 unpacks the key actionable dashboard components: behavioral guidance and risk level visualizations. Both studies examine TPB beliefs and perceived risk as the theoretical mechanisms, uncovering norms to be the key mediator. The work extends the TPB [15] and contributes to health communication and dashboard design.

### 1.1. Psychosocial Beliefs, PHBs, and Dashboards

Extensive TPB research indicates that behavioral change arises in part due to three psychosocial beliefs [22,23]. Attitudes reflect evaluations of the consequences of a behavior. Norms pertain to perceptions that the behavior is appropriate and others are engaging in it. Perceived behavioral control reflects an individual’s belief in their ability to perform the behavior. As a social cognitive theory, TPB emphasizes the role of behavioral intentions. Meta-analyses have shown a moderate correlation (r = 0.48) between these intentions and PHBs [24] and found behavioral intentions to be key in transmitting the effects of TPB psychosocial beliefs on PHB [25].

In the context of the COVID-19 pandemic, Farias and Pilati [19] found that Brazilians reported fewer violations of social distancing mandates when they had stronger attitudes toward the PHB and perceived that friends and family were engaging in the behavior. Similarly, perceiving social distancing or avoiding parties as a norm was positively related to behavioral intentions across several countries [8,20,26]. Research from the United Kingdom [8] and Iran [21] identified perceived behavioral control as a strong predictor of intentions to engage in PHB. Overall, this literature suggests that TPB psychosocial beliefs influence PHBs. However, existing research (based on cross sectional surveys) has not effectively demonstrated how these beliefs can be altered to increase PHB, prompting us to explore a possible health intervention.

#### 1.1.1. Dashboards as a Health Intervention

In the context of the COVID-19 pandemic, researchers have called for interventions “needed to bolster people’s intentions and perceptions of their capacity to perform social distancing behaviors consistently” [8] (p. 1017). Yet, limited attention has been paid to dashboards as intervention tools for health behavior change [27,28]. Dashboards can play a crucial role by transforming data into readable information that conveys trends, patterns, and anomalies [6]. This requires the aggregation and distillation of large amounts of data from a variety of sources. Effective dashboards report key indicators and visualizations that update with timely data snapshots [13]. Researchers have encouraged the adoption of actionable dashboards that present relevant information to the right audience at the right time [12,29,30]. For example, dashboards displaying COVID-19 prevalence on university campuses were deemed essential for safely returning students to campus [10].

Health care experts note that actionable COVID-19 dashboards share characteristics such as geographic case trends, risk levels, and storytelling elements like color coding systems and icons [29,30]. However, a recent review highlights persistent gaps in user engagement and health literacy considerations in dashboard literature, particularly in public health risk communication contexts [12]. Additionally, there is conflicting evidence regarding the impact of health data visualizations on behavior [31,32]. The current research addresses these limitations by examining young adults’ perceptions of dashboards with varying actionability.

#### 1.1.2. Actionable Dashboards and PHBs

Dashboards empower individuals by enabling them to visualize statistics in informative ways [33]. For instance, one study found that implementing a dashboard system for managing type 2 diabetes led to more efficient treatment decisions [34]. Another study found that clinical information dashboards significantly improved medication adherence [35]. Different presentation formats can yield different outcomes; for instance, a ladder format for disease risk was more effective than a bulleted list in increasing physical activity intentions [28]. Additionally, adding explanatory text to vaccination dashboards improved information recall among older adults [14].

Drawing on prior research, we argue that dashboards that clearly communicate the need for action will lead to changes in PHB intentions. Contextualizing data helps individuals comprehend the narrative conveyed by the information [14]. Yet, during the COVID-19 pandemic, few dashboards included such context [12,29,30]. Leveraging prior expert evaluations of dashboards [29,30], we focus on two elements likely to increase actionability: risk level visualization and behavioral guidance. We propose that actionable dashboards, which communicate contextualized virus transmission risk (i.e., risk level visualization) and outline actionable steps to mitigate this risk (i.e., dynamic behavioral guidance), will be more effective than basic dashboards lacking these features. Accordingly, we hypothesize the following:

**H1.**  *Actionable (vs. basic) dashboards will have a positive effect on PHB.*

#### 1.1.3. Dashboards, Psychosocial Beliefs, and PHBs

To understand the causal path of any health intervention, including dashboards, it is essential to examine its impact on intervening beliefs. A meta-analysis found medium effect sizes linking health interventions to TPB psychosocial beliefs [36]. In one study, a video highlighting disapproval from important others changed norms toward safer driving [37]. Compared to basic dashboards, actionable dashboards are expected to increase perceptions that corresponding PHBs are useful (attitudes), appropriate (norms), and feasible (behavioral control). As previously noted, meta-analyses have established associations between TPB psychosocial beliefs and various health behaviors, including smoking cessation, alcohol avoidance, exercise, and healthy eating [24,25,38]. Thus, we anticipate that actionable dashboards highlighting COVID-19 risks and appropriate mitigation strategies will influence TPB psychosocial beliefs.

Perceived risk is another psychosocial belief that may play an intervening role between actionable dashboards and PHB. Health visualizations studies have shown that the presence and framing of risk information significantly influence perceived risk [39]. For instance, presenting death probability from COVID-19 as a frequency (1 in 1000) rather than a percentage (0.1%) increased perceived risk [27].

Based on arguments outlined above, we hypothesize the following:

**H2.**  *Actionable (vs. basic) dashboards will have a significant positive effect on (a) attitudes, (b) behavioral control, (c) norms, and (d) perceived risk.*

**Figure 1:**
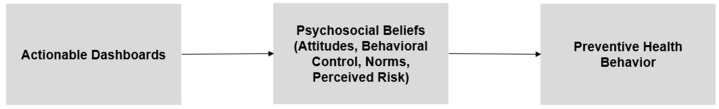
Conceptual model: Actionable dashboards impact preventive health behaviors (PHBs) by influencing psychosocial beliefs.

Figure 1 illustrates our hypotheses in a conceptual model.

### 1.2. Overview of Experimental Studies

To examine the effect of dashboard interventions on psychosocial beliefs and PHB intentions, we conducted two between-subject experimental studies with random assignment to conditions. The studies iteratively tested key actionable dashboard components (dynamic behavioral guidance and risk visualization). Study 1 compared both actionable dashboard components to a basic dashboard displaying the same underlying daily cases. Study 2 isolated the effects of each dashboard component using a 2 (risk level visualizations: no/yes) × 2 (behavioral guidance: no/yes) between-subjects design.

A scenario approach was used to ensure consistency across conditions and to situate both studies during comparable points in the COVID-19 viral waves [40]. In both studies, undergraduate student participants viewed dashboards that displayed COVID-19 cases at a hypothetical university similar to their university in terms of size (medium), pandemic response (COVID-19 dashboard), type (private, urban), and location (eastern United States). Dashboards displayed identical case data across all conditions, representing three days (Monday, Thursday, and Friday) in December (2020 in Study 1; 2021 in Study 2) when COVID-19 cases were rising in the United States and the Centers for Disease Control and Prevention was recommending social distancing and other precautions.

## 2. Materials and Methods—Study 1

### 2.1. Design and Participants

This experiment employed a between-subjects design with two dashboard intervention conditions (basic dashboard and actionable dashboard). The study was approved by the Institutional Review Board at the researchers’ university. During March and April 2021, the SONA system was used to recruit undergraduate students from a medium-sized, private US university to participate in the study. A total of 200 undergraduate students (age *M* = 20.21, *SD* = 1.61; 47.5% male, 51.5% female, 1% self-defined; 21% freshmen, 39.5% sophomores, 29.5% juniors, and 10% seniors; 86.5% White, 5.5% Asian, 4.5% African American, and 1.5% American Indian or Alaska Native; 8% Hispanic or Latino) completed the study for extra credit.

### 2.2. Procedure and Dashboard Intervention

Participants provided their informed consent and interacted with the study materials via the Qualtrics platform. They were asked to imagine being students at a hypothetical university, Blue University. Responses to three reading check questions (e.g., What kind of information about Blue University does the online dashboard display? (a) Times when the dining facilities are busy; (b) Prevalence of COVID-19 on campus; (c) Fundraising at the university; (d) None of the above) suggested that participants understood the task. Only three participants failed to meet the a priori inclusion criterion—answering at least one of the questions correctly. This criterion is desirable because it includes as many participants as possible, while upholding a minimum standard for attention. The remaining 197 participants had been randomly assigned by the Qualtrics platform to the basic condition (*n* = 99) or the actionable condition (*n* = 98).

After being instructed that it was the second week of December 2020, participants were presented with Blue’s COVID-19 dashboard as it appeared on three days. Dashboards displayed the same underlying case data and basic precautions, but the behavioral guidance and risk level visualizations varied between the two conditions.

All participants viewed dashboards that graphed the same new daily student COVID-19 cases during the prior two weeks. Participants viewed a dashboard for Monday 7 December 2020, Thursday 10 December 2020, and Friday 11 December 2020. These dashboards conveyed the same underlying COVID-19 data, which ranged from 0 to 9 new student cases per day with a 7-day rolling average that fluctuated between 1.3 and 2.6 until Monday and then trended upward reaching 5.1 on Thursday and 5.4 on Friday. The dashboards in both conditions also displayed behavioral guidance consistent with the precautions recommended by the U.S. Centers for Disease Control and Prevention at that time. Dashboard images are available via the Open Science Framework (OSF) in the Files section at https://osf.io/x5knw/ (posted on 2 February 2023).

In the basic dashboard intervention condition, the dashboards graphed the count of new cases on campus. Below the graph, there was a list of basic precautions. This behavioral guidance was context independent and, therefore, did not change as a function of the rising case counts.

In the actionable dashboard intervention condition, the dashboards graphed the 7-day rolling average of new cases on campus against the backdrop of a color-coded risk level visualization (e.g., yellow = medium risk, orange = high risk). Monday’s rolling average was at the top of the yellow band, while Thursday’s and Friday’s rolling averages were at the bottom of the orange band. Dashboards also displayed context-dependent behavioral guidance. When the risk level was low on Monday, the dashboard displayed basic precautions. When the risk level increased to high, on Thursday and Friday, the dashboards displayed additional enhanced precautions like “avoid indoor activities” that were reinforced with icons.

### 2.3. Measures

Following guidance and related work in the TPB tradition [15,16], we built our measures by identifying a PHB that was relevant for the population and elicited participants’ behavioral intentions and corresponding psychosocial beliefs. Avoiding crowds and indoor gatherings was a key PHB that public health officials recommended to mitigate the spread of COVID-19 [3]. Failure to adhere to this advice was linked to high COVID-19 infection rates, particularly in young adult populations [2,41]. Socializing is particularly important to young adults, which makes this PHB highly relevant. In accordance with the compatibility principle, intentions to avoid indoor gatherings and parties and the corresponding psychosocial beliefs were measured at the same degree of specificity with respect to context, action, target, and time frame [8,20]. Table 1 describes these measures in detail.

## 3. Results—Study 1

Regression analyses were conducted with IBM SPSS Version 28 to test the hypotheses. In each analysis, we used listwise deletion to exclude participants with missing values. Fear of COVID-19 and gender were included as control variables in all analyses. The dashboard intervention was contrast-coded to reflect whether participants had been assigned to see the actionable dashboard or the basic dashboard (actionable = 0.5, basic = −0.5). As expected, due to random assignment to condition, the control variables, fear of COVID-19 and male gender, did not vary as a function of the dashboard intervention, r = −0.12 and r = −0.08, respectively. Descriptive statistics with *p*-values are shown in Appendix A, Table A1.

**Figure 2:**
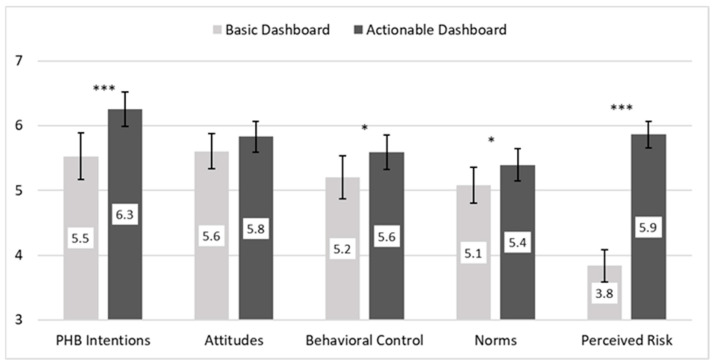
PHB intentions and psychosocial beliefs as a function of the Study 1 dashboard intervention. Note: PHB = preventive health behavior. Bars indicate 95% confidence interval (CI); significance levels come from five analyses of variance (ANOVAs) with actionable dashboard, male gender, and fear of COVID. *** *p* < 0.001; ** *p* < 0.01; * *p* < 0.05.

As shown in Figure 2 (left side), participants who viewed the actionable (vs. basic) dashboard had higher PHB intentions. To test H1, we conducted a hierarchical regression analysis. First, we regressed PHB intentions on the control variables. Consistent with prior research, males had lower PHB intentions, while individuals with greater fear of COVID-19 had higher PHB intentions (see Table 2, Model 1). Next, we regressed PHB intentions on the control variables and the dashboard intervention. The actionable dashboard had a significant positive effect on PHB intentions (see Table 2, Model 2). H1 was supported.

As shown in Figure 2 (middle and right side), psychosocial beliefs were stronger in the actionable dashboard condition compared to the basic condition. To test H2, we regressed each psychosocial belief on the dashboard intervention, gender, and fear of COVID. As shown in Table 3, Section A, the effect of the actionable dashboard on attitudes was marginally significant (*p* = 0.08), failing to support H2a. Compared to the basic dashboard, the actionable dashboard had a statistically significant positive effect on behavioral control (*p* = 0.02), norms (*p* = 0.03), and perceived risk (*p* < 0.001). Hypotheses 2b–d were confirmed.

**Figure 3:**
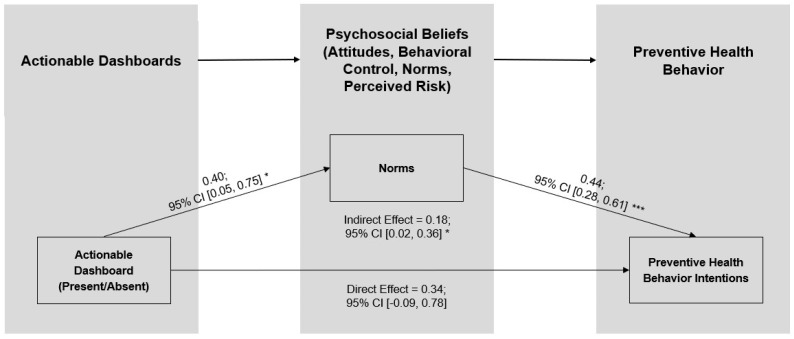
Norms mediate the effect of the Study 1 actionable dashboard on PHB intentions. Note: CI = confidence interval. *** *p* < 0.001; ** *p* < 0.01; * *p* < 0.05.

To explore which of the psychosocial beliefs may explain the effect of the dashboard intervention on PHB intentions, we conducted a parallel mediator analysis [46]. As shown in Table 3, this analysis simultaneously assessed the indirect effect of the actionable dashboard on PHB through each psychosocial belief (i.e., four indirect effect estimates), while controlling for the direct effects of the actionable dashboard on PHB as well as gender and fear of COVID. We used the PROCESS Procedure for SPSS Version 4.2 Model 4 with percentile bootstrap confidence intervals based on 10,000 resamples to estimate an indirect effect for attitudes, B_boot_ = 0.08, SE = 0.07, 95% CI [−0.01, 0.24]; behavioral control, B_boot_ = 0.10, SE = 0.07, 95% CI [−0.01, 0.26]; norms, B_boot_ = 0.18, SE = 0.09, 95% CI [0.02, 0.36]; and perceived risk, B_boot_ = 0.15, SE = 0.17, 95% CI [−0.17, 0.49] (see Table 3, section C). Although the dashboard intervention was associated with each of the psychosocial beliefs (see Table 3, Section A), and three psychosocial beliefs were associated with the PHB (See Table 3, Section B), norms had the only significant indirect effect (see Table 3, Section C). As illustrated in Figure 3, the 95% confidence interval (CI) for the direct effect of the actionable dashboard on PHB includes zero, while the 95% CI for the indirect effect of norms does not, indicating that norms mediated the effect of the dashboard on PHB intentions.

## 4. Discussion—Study 1 and Segue to Study 2

Consistent with H1, presenting the same COVID-19 case data in actionable versus basic dashboards significantly increased young adults’ intentions to engage in the focal PHB, avoiding indoor gatherings and parties. Supportive of H2, participants in the actionable dashboard condition had stronger beliefs that they could perform the PHB (H2b), that the PHB was normative (H2c), and the situation was riskier (H2d). An exploratory analysis that pitted these three beliefs against one another as potential mechanisms revealed that norms uniquely mediated the effect of the dashboard intervention on behavioral intentions.

Study 1 has some limitations. Basic dashboards displayed daily case counts, while the actionable dashboards displayed 7-day rolling averages. Whereas the basic dashboards included a few static recommendations, the actionable dashboards included context-dependent behavioral guidance. When the average cases rose, enhanced precautions were added to match the high-risk-level visualization. Accordingly, the Study 1 actionable dashboards combined behavioral guidance and risk level visualizations. This raises questions about which element may be driving the effect of the dashboard intervention. These limitations were addressed in the second experiment.

Study 2 was conducted to address the key question that arose from Study 1—do actionable dashboards need to have context-dependent behavioral guidance or risk level visualization to promote PHB? As such, Study 2 focuses on the display (or not) of behavioral guidance and the display (or not) of risk level visualizations in actionable dashboards. Of these two components, we expect that behavioral guidance is likely to be the most important. It helps people make sense of the recommended action and is more closely linked to storytelling [30]. Risk level visualizations could scare people and lead them to take riskier actions, especially if they feel that the situation is out of control and their actions would be futile [47]. Risk perceptions tend to be a less influential (or insignificant) driver of behavior when TPB beliefs are considered [8,48]. We observed a similar pattern in Study 1, with TPB beliefs—but not risk—associated with PHB intentions (see Table 3, Section B). Furthermore, the Study 1 finding that norms (but not risk) mediated the effect of the actionable dashboard intervention lends support to the focus on the behavioral guidance component. Accordingly, we examine the following Study 2 hypotheses.

**H3.**  *Behavioral guidance will have a positive effect on PHB.*

**H4.**  *Behavioral guidance will have a positive effect on (a) attitudes, (b) behavioral control, and (c) norms.*

**H5.**  *Norms will mediate the effect of behavioral guidance on PHB.*

## 5. Materials and Methods—Study 2

### 5.1. Design and Participants

Study 2 used a 2 (risk level visualization: yes/no) × 2 (behavioral guidance: yes/no) between-subjects dashboard intervention design. Accordingly, Study 2 participants were randomly assigned to view dashboards that graphed the same average daily cases and showed (a) no additional information, (b) risk assessment, (c) behavioral guidance, or (d) risk assessment and behavioral guidance. All dashboards displayed the 7-day rolling average of the same daily cases. The context-dependent risk level visualization was manipulated by including or not including a risk label (moderate/high) and a corresponding color-coded risk scheme (yellow/orange) in the background of the rolling average case graph. Dynamic, context-dependent behavioral guidance was manipulated by including or not including data-driven precautions via text and simple icons (e.g., avoid indoor activities). The study was approved by the Institutional Review Board at the researchers’ university.

During March and April 2022, the SONA system was used to recruit a sample of undergraduate students to participate in the study from the same university as in Study 1. Respondents were 228 students (age *M* = 20.09, *SD* = 2.10; 46.1% male, 53.9% female; 0% non-binary; 34.2% freshmen, 32.9% sophomores, 24.1% juniors, and 8.3% seniors; 91.2% White, 5.3% African American, 2.2% Asian, and 4.8% Hispanic or Latino), who completed the study for extra credit. Responses to three reading check questions suggested that the task was understood by participants [49]. None of the participants failed to meet the a priori inclusion criteria, answering at least one question correctly. The 228 participants were randomly assigned to one of the four dashboard intervention conditions.

### 5.2. Procedure and Dashboard Interventions

**Figure 4:**
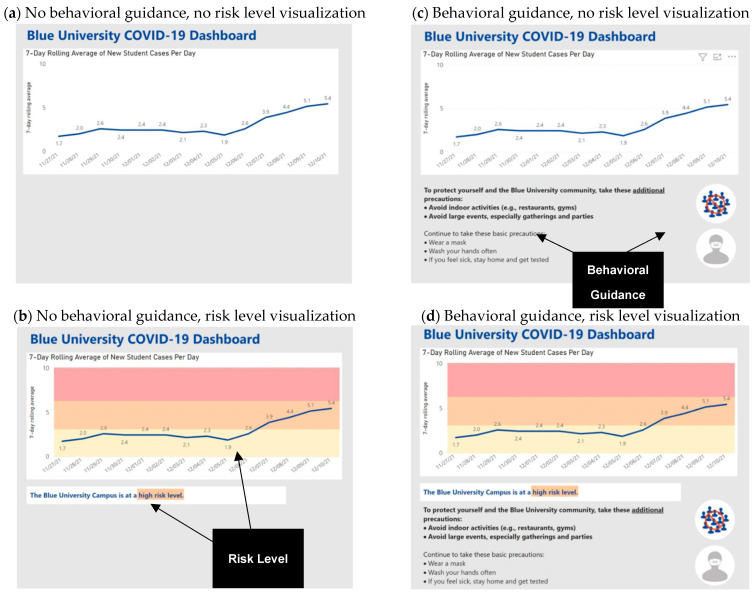
Dashboard interventions with annotations.

Participants provided their informed consent and interacted with study materials via Qualtrics. As in Study 1, participants were asked to imagine they were students at Blue University. Participants were shown three images of Blue’s COVID-19 dashboard, one for Monday, 6 December 2021, one for Thursday, 9 December 2021, and one for Friday, 10 December 2021. Each dashboard graphically displayed new student cases for the prior two weeks with Monday’s dashboard showing daily cases since 23 November 2021 and Friday’s showing new cases since 27 November 21. As in Study 1, daily COVID-19 cases showed an upward trend, with the seven-day rolling average starting at 2.6 on Monday and ending at 5.1 and 5.4 on Thursday and Friday, respectively. The intervention varied in whether dashboards that graphically displayed the same rolling average cases also included one of the two actionable dashboard components shown in Figure 4 and detailed next.

Risk level visualization. The risk level visualization included a risk statement below the graph (e.g., “Blue University is at a moderate risk level”) and a corresponding color-coded risk scheme in the background of the rolling average case graph. From 23 November 2021 until 6 December 2021, the rolling average fluctuated between 1.3 and 2.6 and was plotted in the yellow, moderate risk band. As the rolling average moved above 2.6, the risk level moved from moderate (i.e., yellow) on Monday to high (i.e., orange) on Thursday and Friday.

Behavioral guidance. The behavioral guidance included basic, context-independent precautions (e.g., wear a mask) and enhanced, context-driven precautions. As the rolling average cases increased above the threshold between moderate and high risk, the behavioral guidance changed too. On Monday, when the rolling average was below 2.6 cases, the dashboard only included context-independent precautions. On Thursday and Friday, when the rolling average moved above 2.6 cases, the dashboard also displayed enhanced precautions (e.g., avoid large events) and a no-crowd icon to reinforce the precautions.

Figure 4 shows how the risk level visualization and behavioral guidance conditions cross in the 2 × 2 between-subjects factorial study design. This figure also shows annotations highlighting the actionable dashboard components. The condition that included behavioral guidance and risk level visualizations is the most comparable to the Study 1 actionable dashboard intervention. Dashboard images are available in the Files section of the project OSF at https://osf.io/x5knw/ (posted on 2 February 2023).

### 5.3. Measures

Similar to Study 1, we focused on the effect of the dashboard intervention on our focal PHB: intentions to avoid indoor gatherings and parties. Behavioral guidance and risk level visualization were contrast-coded to reflect whether participants had been randomly assigned to see each actionable dashboard component (behavioral guidance: yes = 0.5, no = −0.5; risk level visualization: yes = 0.5, no = −0.5). Participants reported their current gender as male, female, non-binary, or prefer not to answer. Otherwise, all measures and scales were identical to Study 1 (see Table 1).

## 6. Results—Study 2

We followed an analytical approach comparable to Study 1. Fear of COVID-19 and gender were included as control variables in all analyses, which were conducted in SPSS Version 28. Again, we used listwise deletion for missing values. As expected, the control variables, fear of COVID-19 and male gender, were not significantly associated with behavioral guidance (r = −0.04 and r = −0.06, respectively) or risk level visualization (r = −0.04 and r = 0.01, respectively). Descriptive statistics with *p*-values are shown in Appendix A, Table A2.

**Figure 5:**
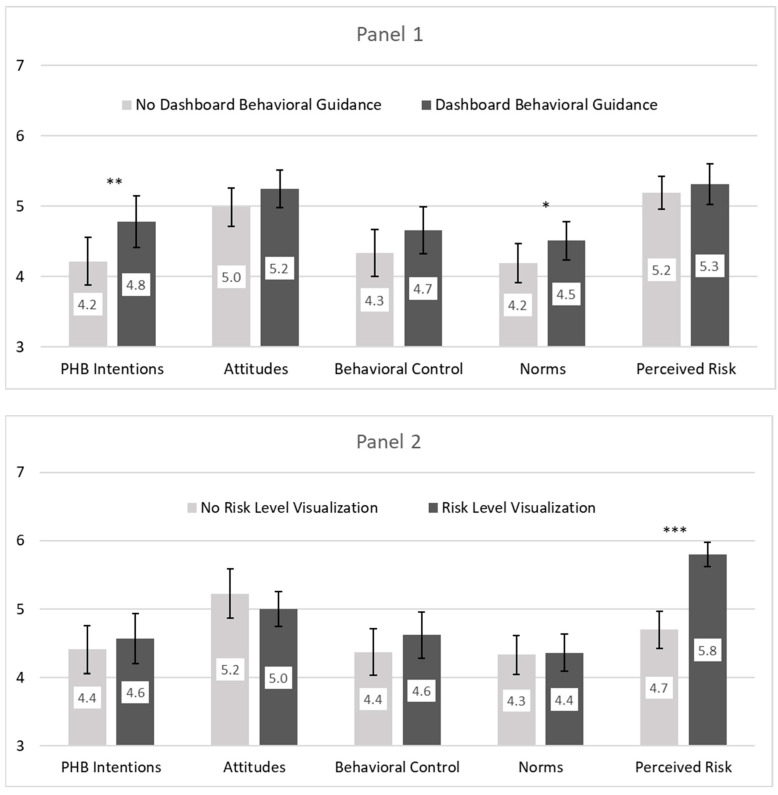
PHB intentions and psychosocial beliefs as a function of the Study 2 dashboard behavioral guidance (panel 1) and risk level visualization (panel 2). Note: PHB = preventive health behavior. Bars indicate 95% CI; significance levels come from five analyses of variance (ANOVAs) with behavioral guidance, risk visualization, their interaction, male gender, and fear of COVID. *** *p* < 0.001; ** *p* < 0.01; * *p* < 0.05.

Participants who viewed dashboards with behavioral guidance had significantly higher PHB intentions (see Figure 5, Panel 1), while those who viewed dashboards with risk level visualizations did not have higher PHB intentions, though they perceived significantly greater risk (see Figure 5, Panel 2). As in Study 1, the first hierarchical regression with control variables showed males with lower PHB intentions (see Table 4, Model 1). The second regression revealed that behavioral guidance had a significant positive effect on PHB intentions (see Table 4, Model 2), confirming H3.

To test H4, we regressed each psychosocial belief on behavioral guidance, risk level visualization, their interaction, gender, and fear of COVID. As shown in Table 5, Section A, there was no significant effect of behavioral guidance on attitudes (*p* = 0.15) or behavioral control (*p* = 0.12); therefore, H4a and H4b were not supported. Behavioral guidance did have a significant effect on norms (*p* = 0.04), confirming H4c.

**Figure 6:**
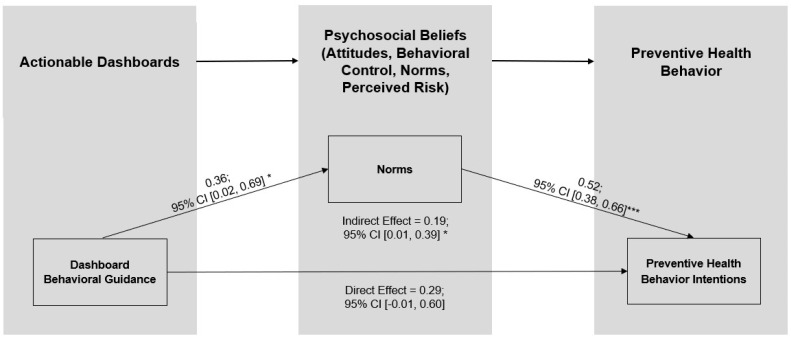
Norms mediate the effect of the Study 2 dashboard guidance on PHB intentions. Note: CI = confidence interval. *** *p* < 0.001; ** *p* < 0.01; * *p* < 0.05.

To test H5, we conducted a parallel mediator analysis that simultaneously examined each psychosocial belief as a potential mediator. We used PROCESS Version 4.2 Model 4 with percentile bootstrap confidence intervals based on 10,000 resamples (see Table 5, section C). As hypothesized and shown in Figure 6, we found a significant indirect effect of norms, B_boot_ = 0.19, SE = 0.10, 95% CI [0.01, 0.39]. H5 was supported.

Results provide insight into what components of actionable dashboards alter young adults’ beliefs and in turn their intentions to engage in PHB. Consistent with H3, results show that presenting the exact same graphs of rolling average COVID-19 case data with context-dependent behavioral guidance significantly increases young adults’ PHB intentions. No such effect on PHB was found for the inclusion of a context-dependent risk level visualization.

Supportive of H4c, participants in the behavioral guidance condition believed the PHB to be significantly more normative than did their peers who viewed dashboards with no behavioral guidance. Consistent with H5, norms uniquely mediated the effect of the behavioral guidance component of the actionable dashboard intervention on PHB, highlighting the importance of norms as an intervening psychosocial belief.

## 7. Discussion

### 7.1. Actionable Dashboards and the Importance of Behavioral Guidance

This research leverages insights from the data sciences [30] and the TPB [22] to examine whether and how actionable dashboards influence PHB. Researchers have noted the key role digital communications play in empowering individuals during epidemic outbreaks and called for studies to examine target audiences’ perceptions of actionable dashboards [30]. Our findings extend understanding of effective dashboards beyond what had previously been gleaned from case studies and expert categorizations [6,30,33]. Our research used a theoretically grounded experimental design with between-subjects random assignment of the actionable dashboard components to examine how dashboards influence PHB intentions.

In Study 1, actionable dashboards significantly increased intentions to engage in PHBs compared to basic dashboards. While basic dashboards provide transparency through daily case updates, their lack of contextual framing can make data interpretation challenging [27]. In contrast, actionable dashboards offer context through risk level visualizations and suggest specific actions via behavioral guidance. Study 2 pitted components of actionable dashboards against each other and found that including behavioral guidance enhances PHB intentions, while risk level visualizations alone did not yield significant effects. These findings underscore the importance of behavioral guidance in managing high-risk situations, as it helps individuals perceive risks as manageable and directs them toward appropriate PHBs. Community dashboard designers should prioritize incorporating relevant behavioral guidance aligned with various health contexts, such as infectious disease management, air quality monitoring, and drinking water safety. Recent work illustrates how Internet of Things-based dashboards can estimate and visualize COVID-19 aerosol transmission risk in indoor environments, offering a compelling example of how technical systems can support behavior change through real-time environmental monitoring [50]. However, their system does not explicitly address psychosocial mechanisms—such as social norms—which our findings highlight as critical drivers of PHB intentions.

### 7.2. Actionable Dashboards, TPB Psychosocial Beliefs, and the Mediating Role of Norms

Our work heeds the call to systematically investigate how TPB elements can be harnessed for effective online health communication targeted at digitally savvy young adults [8,9]. Across both studies, norms emerged as a key psychosocial belief influenced by dashboard interventions, which in turn affected PHB intentions. This result aligns with correlational evidence indicating that individuals are less likely to violate COVID-19 social distancing mandates when they believe their close peers and family members are adhering to these PHBs [19]. During a pandemic, deciphering the appropriate actions amidst the abundance of public health data and communications is a significant challenge [51]. Social norms are crucial in helping individuals navigate novel and uncertain situations, guiding them to interpret public health data accurately and make informed decisions [52]. Our studies demonstrate that actionable dashboards assist young adults in navigating complex health information by providing normative behavioral guidance, ultimately increasing their intentions to engage in PHBs.

### 7.3. Limitations and Future Research

While our studies effectively establish causal relationships between dashboard components and PHB intentions, they also present trade-offs between experimental control and generalizability. Randomly exposing participants to different dashboard conditions allowed us to draw causal inferences, but it limited our ability to measure actual behavior rather than just intentions. Although meta-analyses reveal such intentions significantly predict health behaviors [24,25,38], this remains a limitation. Social distancing PHB requires not engaging in certain behaviors, which regulatory focus theory suggests triggers an avoidance, rather than an approach, orientation [53]. It may be that actionable dashboards generalize to support avoidance PHB, such as not exercising outside when the air quality index is high, more than to support approach PHB, such as swimming in a lake when bacterial levels are low.

This study tested the effectiveness of actionable dashboard interventions in young adults. Disparities in health motivation, efficacy, and knowledge between younger and older adults [54], along with variations in disease vulnerability and perceived risk, underscore the need for further investigation into how dashboard interventions impact age groups differently. Furthermore, cultural factors may influence the relationship between norms and behavioral intentions; prior research indicates that power distance affects this relationship [55]. Given that our sample comes from the United States, characterized by moderate power distance, future research could test our dashboard interventions across cultural contexts to enhance understanding of norms and PHB intentions globally. Lastly, exploring how digital dashboards can promote health equity by addressing social determinants of health will be vital [56]. By ensuring data transparency and providing real-time information on pandemic hotspots and recommended responses dashboards can empower all communities, particularly marginalized groups, to make informed decisions regarding social distancing and other PHBs.

## 8. Conclusions

The present work makes several contributions. First, it offers a theoretical framework that extends the TPB to examine the differential impact of two dashboard components—risk level visualizations and behavioral recommendations—on young adults. Second, our studies overcome the limitations of prior correlational studies examining TPB psychosocial beliefs and PHB by using randomized between-subjects experimental designs. Third, we uncover the importance of social norms in mediating the effect of dashboard behavioral guidance on PHB. Finally, our findings provide valuable insights for researchers, public health experts, and policymakers in designing effective public-facing dashboards that communicate health risks.

## Figures and Tables

**Table 1:** Study 1 and Study 2 measures.

Measure	Description	Scale	Cronbach’s α or *r*
PHB Intentions (DV)	(a) I will make an effort/(b) I will try to avoid indoor gatherings and parties during the next week [15,16,42]	(a) 1 = definitely false to 7 = definitely true, (b) 1 = definitely will not to 7 = definitely will	0.97
Attitudes	My avoiding indoor gatherings and parties during the next week would be ___ [43]	(a) 1 = harmful, 7 = beneficial, (b) 1 = stressful, 7 = relaxing, (c) 1 = foolish, 7 = wise; and (d) 1 = bad, 7 = good	0.84
Behavioral Control	(a) For me, to avoid indoor gatherings and parties during the next week will be __ and (b) I am confident that I can avoid indoor gatherings and parties during the next week [43]	(a) 1 = very difficult, 7 = very easy, (b) 1 = strongly disagree, 7 = strongly agree	0.84
Norms	(a) Most of my friends/(b) Most of my family would avoid indoor gatherings and parties, (c) People who are important to me would _____ of my avoiding indoor gatherings and parties over the next week [43]	(a) and (b) 1 = strongly disagree, 7 = strongly agree, (c) 1 = disapprove, 7 = approve	0.73
Perceived Risk	(a) The risk of getting COVID-19 at Blue University is __, (b) COVID-19 on Blue’s campus is __	(a) 1 = low risk, 7 = high risk, (b) 1 = under control, 7 = out of control	0.83
Fear of COVID-19 (control ^1^)	(a) I am most afraid of the coronavirus, (b) It makes me uncomfortable to think about coronavirus, (c) I am afraid of losing my life because of the coronavirus, and (d) When watching news and stories about the coronavirus on social media, I become nervous or anxious [44,45]	1 = strongly disagree, 7 = strongly agree	0.88
Male gender (control ^2^)	Asked current gender as male, female, self-defined, or prefer not to answer options	Contrast coded 0.5 = male and −0.5 = non-male	n/a

^1^ We control for this emotional reaction because it was found to correlate to social distancing compliance [42]. ^2^ We control for this individual difference because engaging in the discretionary PHB may signal vulnerability and go against gender stereotypes. Survey research revealed lower compliance with COVID-19 social distancing mandates among male than among female respondents [8,19].

**Table 2:** Regression of Study 1.

Variable	Model 1			Model 2		
	*B*	*SE*	*p*	*B*	*SE*	*p*
Constant	5.13 ***	0.24	<0.001	5.01 ***	0.23	<0.001
Male gender	−0.54 *	0.23	0.019	−0.43	0.22	0.06
Fear of COVID-19	0.24 ***	0.07	<0.001	0.28 ***	0.07	<0.001
H1: Actionable dashboard				0.84 ***	0.21	<0.001
*R* ^2^	0.12			0.19		
△*R*^2^				0.07 ***		

Note: *** *p* < 0.001; ** *p* < 0.01; * *p* < 0.05.

**Table 3:** Model coefficients for PROCESS parallel mediator analysis in Study 1.

	*B*	*p*	LLCI	ULCI
A. Effects of Dashboard Intervention on Psychosocial Beliefs				
Attitudes (*a*_1_)	0.304	0.081	−0.037	0.645
Behavioral control (*a*_2_)	0.501 *	0.018	0.089	0.913
Norms (*a*_3_)	0.399 *	0.026	0.047	0.751
Perceived risk (*a*_4_)	2.072 ***	0.000	1.754	2.390
B. Effects of Psychosocial Belief on PHB Intentions				
Attitudes (*b*_1_)	0.257 **	0.004	0.081	0.433
Behavioral control (*b*_2_)	0.196 **	0.005	0.061	0.332
Norms (*b*_3_)	0.444 *	0.000	0.279	0.609
Perceived risk (*b*_4_)	0.071	0.342	−0.076	0.218
C. Indirect Effects of Dashboard Intervention via the Competing Psychosocial Beliefs				
Attitudes (*c*_1_)	0.078	-	−0.010	0.244
Behavioral control (*c*_2_)	0.098	-	−0.008	0.260
**Norms** (***c*_3_**)	**0.177**	**-**	**0.017**	**0.364**
Perceived risk (*c*_4_)	0.147	-	−0.174	0.493

Note: LLCI = lower limit of confidence interval, ULCI = upper limit of confidence interval, PHB = preventive health behavior. Letters next to psychosocial belief refer to the paths in the PROCESS model. Bold text highlights the indirect effects with a 95% CI that does not include 0, indicating a statistically significant mediation effect [46]. * *p* < 0.05; ** *p* < 0.01; *** *p* < 0.001.

**Table 4:** Regression of Study 2.

Variable	Model 1			Model 2		
	*B*	*SE*	*p*	*B*	*SE*	*p*
Constant	2.88 ***	0.23	<0.001	2.85 ***	0.23	<0.001
Male gender	−0.48 *	0.23	0.04	−0.43	0.22	0.06
Fear of COVID	0.59 ***	0.07	<0.001	0.60 ***	0.07	<0.001
H3: behavioral guidance				0.61 **	0.21	0.005
Risk level visualization				0.22	0.21	0.29
Interaction of BG and RLV				−0.31	0.42	0.47
*R* ^2^	0.27			0.30		
△*R*^2^				0.03 *		

Note: BG = behavioral guidance; RLV = risk level visualization. *** *p* < 0.001; ** *p* < 0.01; * *p* < 0.05.

**Table 5:** Model coefficients for PROCESS parallel mediator analysis in Study 2.

	*B*	*p*	LLCI	ULCI
A. Effects of Dashboard Intervention on Psychosocial Beliefs				
Attitudes (*a*_1_)	0.264	0.149	−0.095	0.623
Behavioral control (*a*_2_)	0.349	0.120	−0.092	0.790
Norms (*a*_3_)	0.355 *	0.040	0.016	0.694
Perceived risk (*a*_4_)	0.111	0.488	−0.203	0.434
B. Effects of Psychosocial Belief on PHB Intentions				
Attitudes (*b*_1_)	0.030	0.666	−0.105	0.164
Behavioral control (*b*_2_)	0.257 ***	0.000	0.147	0.368
Norms (*b*_3_)	0.522 ***	0.000	0.380	0.664
Perceived risk (*b*_4_)	0.295 ***	0.000	0.161	0.428
C. Indirect Effects of Dashboard Intervention via the Competing Psychosocial Beliefs				
Attitudes (*c*_1_)	0.008	-	−0.041	0.067
Behavioral control (*c*_2_)	0.090	-	−0.023	0.222
**Norms** (***c*_3_**)	**0.185**	**-**	**0** **.009**	**0** **.387**
Perceived risk (*c*_4_)	0.033	-	−0.060	0.143

Note: LLCI = lower limit of confidence interval, ULCI = Upper limit of confidence interval, PHB = preventive health behavior. Letters next to psychosocial belief refer to the paths in the PROCESS model. Bold text highlights the indirect effects with a 95% CI that does not include 0, indicating a statistically significant mediation effect [46]. * *p* < 0.05; ** *p* < 0.01; *** *p* < 0.001.

## Data Availability

The data, code book data dictionaries, syntax and results upon which conclusions are based for Study 1 and Study 2 are openly available in OSF at https://doi.org/10.17605/OSF.IO/4JFU8 (registered on 10 April 2025).

## Abbreviations

The following abbreviations are used in this manuscript:

PHB	Preventive health behavior
TPB	Theory of planned behavior

## Appendix A

**Table A1:** Mean, standard deviation, and correlations of Study 1 variables.

		*M*	*SD*	1	2	3	4	5	6	7	8
1	Actionable dashboard	0.00	0.50	-							
2	Male gender	−0.02	0.50	−0.08	-						
3	Fear of COVID	3.18	1.70	−0.12	−0.32 ***	-					
4	Attitudes	5.72	1.27	0.09	−0.21 **	0.35 ***	-				
5	Behavioral control	5.40	1.52	0.12	−0.14	0.31 ***	0.61 ***	-			
6	Norms	5.24	1.32	0.12	−0.22 **	0.33 ***	0.63 ***	0.53 ***	-		
7	Perceived risk	4.83	1.53	0.67 ***	−0.11	0.04	0.17 *	0.11	0.29 ***	-	
8	PHB intentions	5.89	1.62	0.22 **	−0.25 ***	0.32 ***	0.60 ***	0.55 ***	0.66 ***	0.31 ***	-

Note. *n* = 197. Abbreviations: PHB = preventive health behavior; COVID-19 = coronavirus disease 2019. * *p* < 0.05. ** *p* < 0.01. *** *p* < 0.001. Significance levels should be interpreted cautiously. The proper error terms are applied in the analyses in Table 2 and Table 3.

**Table A2:** Mean, standard deviation, and correlation of Study 2 variables.

		*M*	*SD*	1	2	3	4	5	6	7	8	9
1	Behavioral guidance	−0.02	0.50	-								
2	Risk level visualization	0.00	0.50	0.02	-							
3	Male	−0.04	0.50	−0.06	0.01	-						
4	Fear of COVID	2.71	1.52	−0.04	−0.04	−0.30 ***	-					
5	Attitudes	5.11	1.43	0.09	−0.08	−0.21 **	0.28 ***	-				
6	Behavioral control	4.49	1.78	0.09	0.07	−0.16 *	0.32 ***	0.54 ***	-			
7	Norms	4.35	1.47	0.11	0.01	−0.25 ***	0.46 ***	0.53 ***	0.58 ***	-		
8	Perceived risk	5.25	1.34	0.05	0.41 ***	−0.13	0.18 **	0.27 ***	0.22 **	0.31 ***	-	
9	PHB intentions	4.49	1.90	0.15 *	0.04	−0.27 ***	0.51 ***	0.50 ***	0.61 ***	0.73 ***	0.41 ***	-

Note. *n* = 228. Abbreviations: PHB = preventive health behavior; COVID-19 = coronavirus disease 2019. * *p* < 0.05. ** *p* < 0.01. *** *p* < 0.001. Significance levels should be interpreted cautiously. The proper error terms are applied in the analyses in Table 4 and Table 5.
